# Central Serous Chorioretinopathy and Ocular Comorbidities

**DOI:** 10.3390/jcm14030720

**Published:** 2025-01-23

**Authors:** Anindya Samanta, Matthew Driban, Niroj Sahoo, Deepika Parameswarappa, Sumit Randhir Singh, Sonny Caplash, Pranjal Mishra, Rohit Agrawal, Ramesh Venkatesh, Dmitrii S. Maltsev, Jay Chhablani

**Affiliations:** 1 Department of Ophthalmology, Baylor College of Medicine, Houston, TX 77030, USA; 2 UPMC Eye Center, University of Pittsburgh, Pittsburgh, PA 15261, USA; driban.matthew@medstudent.pitt.edu (M.D.); caplashs@upmc.edu (S.C.); jay.chhablani@gmail.com (J.C.); 3 LV Prasade Eye Institute, Kallam Anji Reddy Campus, LV Prasad Marg, opp. PVR, Park View Enclave, Banjara Hills, Hyderabad 500034, India; nirojsahoo71@gmail.com (N.S.); deepikacpd@gmail.com (D.P.); 4 Department of Vitreoretina, Akhand Jyoti Eye Hospital, CoE Mastichak, Saran 841219, India; sumit.jipmer@gmail.com; 5 Narayana Nethralaya, 121/C Chord Road, 1st ‘R’ Block, Rajaji Nagar, Bangalore 560010, India; p.mishra.doc@gmail.com (P.M.); agrawaldrrohit@gmail.com (R.A.); vramesh80@yahoo.com (R.V.); 6 Military Medical Academy, 21, Botkinskaya Str., St. Petersburg 194044, Russia; glaz.med@yandex.ru

**Keywords:** Central serous chorioretinopathy, ocular comorbidities, retinal detachment, CSCR

## Abstract

**Background**/**Objectives**: Central serous chorioretinopathy (CSCR) is a common retinopathy that can present with other concurrent diseases; thus, further research into the prevalence of other ocular comorbidities in eyes with CSCR is required. **Methods**: This retrospective, multicentric, cross-sectional observational study reviewed the charts of 9157 patients. Of them, 579 (6.32%) patients and 766 eyes had an additional ocular comorbidity, in addition to CSCR, in at least one subject eye. **Results**: The baseline best-corrected visual acuity (BCVA) of the subjects eyes was 0.49 ± 0.36 logMAR. The average BCVA of subject eyes with coexisting macular diseases was 0.50 logMAR, while the corresponding BCVA of subject eyes with coexisting peripheral disease was 0.55 logMAR. The most prevalent coexisting macular diseases were non-proliferative diabetic retinopathy (26.8%), non-exudative age-related macular degeneration (AMD) (7.6%) and hypertensive retinopathy (3.0%). The most prevalent coexisting non-macular diseases were lattice degeneration (8.9%), optic atrophy (5.1%), rhegmatogenous retinal detachment (1.70%) and optic disc pit (1.7%). The odds of having a comorbid disease in the same eye as CSCR were statistically significant for branch retinal vein occlusion (OR 11.56, *p*-value = 0.02) and non-exudative AMD (OR 2.06; *p*-value = 0.01); additionally, there was a trend towards significance for idiopathic polypoidal choroidal vasculopathy (OR 4.43; *p*-value = 0.05) when compared to the eyes without CSCR. **Conclusions**: Certain diseases are more likely to coexist in eyes with CSCR. Additionally, eyes with CSCR may have statistically significant odds of certain diseases when compared to eyes without CSCR.

## 1. Introduction

Central serous chorioretinopathy (CSCR) is the fourth most common retinopathy after age-related macular degeneration (AMD), diabetic retinopathy (DR) and branch retinal vein occlusion (BRVO) [1,2]. It primarily affects males between the ages of 28 to 68 years. Risk factors for CSCR have previously included age, sex, pregnancy, antibiotic use, alcohol, hypertension, obstructive sleep apnea, endogenous and exogenous steroids, systemic lupus erythematous, organ transplant and end stage renal disease [3]. However, there have been large-scale studies that provide, at times, a mixed picture in regard to the potential risk factors of CSCR. A recent case control study found steroids, Cushing syndrome and diabetes mellitus to be more likely in patients with CSCR [4]. Importantly, this study found that patients with previously reported factors including hypertension, pregnancy and sleep disorders have lower odds of having CSCR. Other studies have found that macular edema, AMD, is more associated with developing CSCR, while glaucoma and proliferative diabetic retinopathy (PDR) were less associated with CSCR [5].

CSCR can have overlapping features with other ocular pathologies, as well as being present alongside other ocular diseases. Previous studies have shown that there is a coexistence of CSCR with macular telangiectasia, DR and polypoidal choroidal vasculopathy [6,7,8,9]. The management of CSCR and associated ocular diseases could be different or complementary. Furthermore, there are no large database studies in the literature documenting the frequency of associated/coexistent ocular diseases with CSCR.

The purpose of this study is twofold. Firstly, we aimed to determine the prevalence of different ocular comorbidities in eyes with CSCR. Secondly, we aimed to determine whether ocular comorbidities that are present in CSCR patients are more likely to occur in the eye with CSCR (subject eye) when compared to the other eye without CSCR (fellow eye).

## 2. Materials/Subjects and Methods

This was a retrospective, multicentric, cross-sectional observational study. Institutional review board (IRB) approval was obtained from all of the participating centers (STUDY20030204, protocol #255 and BHR-R-12-20-564). The study adhered to the principles of the Declaration of Helsinki and was approved by the respective institutional research boards. Data of all patients with an ocular diagnosis of CSCR were retrieved from the electronic medical records from January 2016 to December 2020. The retrospective data were deidentified and, as such, informed consent was not required for the study. The electronic medical records system had a pre-formatted template, which was filled by trained ophthalmic technicians and ophthalmologists during patients’ visits to the clinic. Apart from querying the International Classification of Diseases (ICD) 9th revision coding of CSCR, all patient records underwent a thorough keyword search of “CSCR”, “CSR” and “Central serous chorioretinopathy” from the systemic history, retina evaluation and plan of management notes. Patients were similarly classified as having another ocular comorbidity based on ICD-9 codes. Exclusion criteria included patients with missing information about vision, clinical findings, the wrong code for CSCR and a final diagnosis that was not CSCR. At each site, for their initial visit, patients underwent a comprehensive ophthalmic evaluation including medical history, best-corrected visual acuity (BCVA), measurement of intraocular pressure, anterior segment slit-lamp biomicroscopy and dilated fundus examination. Findings in the posterior dilated fundal exam were noted and compiled for each patient.

## 3. Statistical Analysis

A univariate analysis was conducted for the various characteristics at baseline. An OR with a 95% confidence interval (CI) was calculated for each ocular comorbidity. Non-paired student’s *t*-tests were performed to calculate the *p*-value, wherein a *p*-value of less than 0.05 was considered significant.

## 4. Results

Our study reviewed the charts of 9157 patients with CSCR, from four study centers. Eyes with additional comorbidities were listed as the subject eye. For patients with presentation of CSCR in only one eye, the other eye was recorded as a fellow eye. For patients with presentation of CSCR in both eyes, both eyes were recorded as subject eyes.

Table 1 shows the baseline demographic data of patients in this study. Patients without comorbid ocular findings alongside CSCR (93.68%) were excluded from the study. A total of 579 (6.32%) patients and 766 eyes were included in the study. The average age of the patients was 50.3 ± 13.1 years, with a 4:1 male to female ratio. The most common systemic diseases were diabetic mellitus (34.9%) and hypertension (14.9%). Additionally, 18 patients (3.1%) had autoimmune diseases.

**Figure 1:**
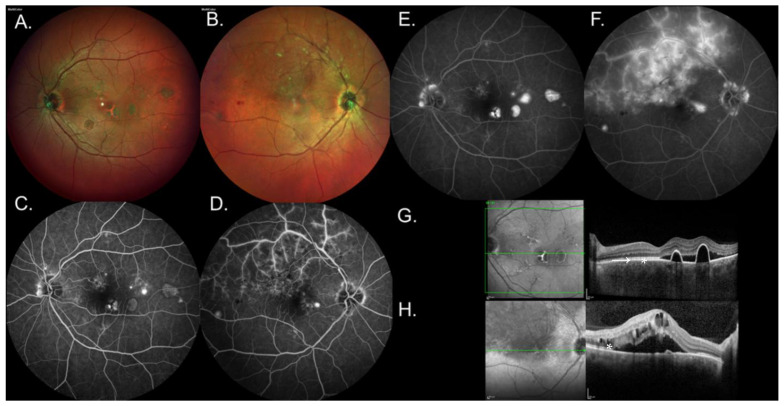
Images of a patient with central serous chorioretinopathy (CSCR) in both eyes and branch retinal vein occlusion in the left eye. Visual acuity of the patient was 0.30 and 0 logMAR in the right and left eye, respectively. (**A**,**B**) show fundus photography of the right and left eye, respectively. (**A**) shows pigmentary changes and multiple areas of atrophic change. (**B**) has an ischemic superior fundus with cotton-wool spots surrounding the superior arcades. (**C**,**D**) show the arteriovenous phase of fluorescein angiogram (FA) of the right and left eye, respectively. (**E**,**F**) show the late phase of FA of the right and left eye, respectively. (**C**,**E**) show pooling of the dye in temporal fovea, characteristic of CSCR and window defects. (**D**,**F**) show pooling temporally and leakage from the superior vessels. (**G**,**H**) show optical coherence tomography of the right and left eye, respectively. (**G**) shows subretinal fluid (*) and two pigment epithelial detachments (PED) (→). (**H**) shows cystoid macular edema (*).

**Figure 2:**
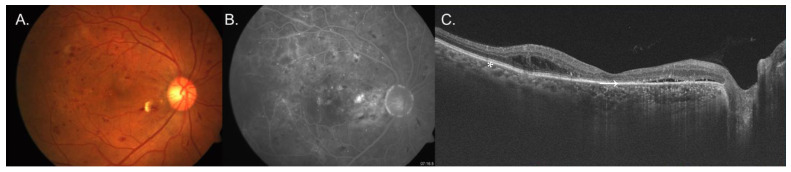
Images of a patient with central serous chorioretinopathy (CSCR) and diabetic retinopathy (DR) in the right eye. (**A**) shows fundus photography with cystoid macular edema temporally, multiple microaneurysms and a dot blot hemorrhage in the posterior pole. (**B**) shows a fluorescein angiogram with the focal area of petaloid leakage. (**C**) shows optical coherence of the same eye with intraretinal cystoid macular edema (*) and subretinal fluid (→).

**Figure 3:**
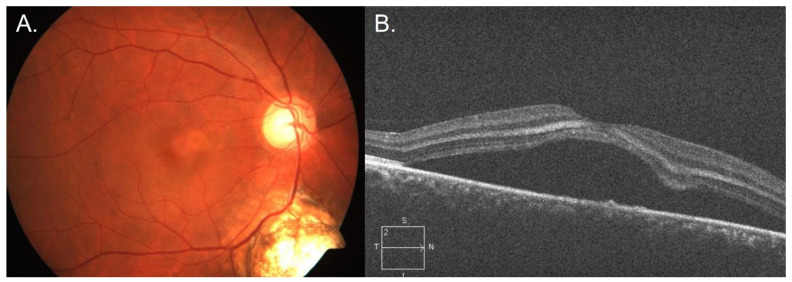
Images of a patient with central serous chorioretinopathy (CSCR) and a choroidal coloboma in the left eye. (**A**) shows fundus photography of the left eye with a cystoid macular edema temporally and a coloboma inferiorly. (**B**) shows optical coherence tomography of the left eye with a large pigment epithelial detachment.

Table 2 shows the prevalence of concurrent diseases in addition to CSCR in the same eye. It also shows the prevalence of disease without CSCR in the fellow eye. Figure 1 shows an example of a patient with CSCR in both eyes and a BRVO in one eye. Figure 2 shows an example of a patient with concurrent CSCR and DR. Figure 3 shows an example of a patient with concurrent CSCR and coloboma.

There was a difference (0.35 logMAR) in the BCVA between eyes that had ocular comorbidity and the fellow eyes. The average BCVA of eyes with coexisting macular diseases listed in Table 2 was 0.50 logMAR, while the corresponding BCVA of eyes with coexisting peripheral disease was 0.55 logMAR. The most prevalent coexisting macular diseases were non-proliferative diabetic retinopathy (26.8%), non-exudative age-related macular degeneration (AMD) (7.6%) and hypertensive retinopathy (3.0%). The most prevalent coexisting non-macular diseases were lattice degeneration (8.9%), optic atrophy (5.1%), rhegmatogenous retinal detachment (1.7%) and optic disc pit (1.7%).

CSCR may increase the odds of having certain concurrent findings. In this study, there was a statically significant odds ratio (OR) for branch retinal vein occlusion (OR 11.56, *p* = 0.02), non-exudative AMD (OR 2.06; *p* = 0.01) and peripheral pigmentary changes (OR 2.72; *p* = 0.03). There was a trend towards significance for the OR of idiopathic polypoidal choroidal vasculopathy (OR 4.43; *p* = 0.05) in the subject eye.

In our study, three patients had concurrent panuveitis in both eyes, and they were subsequently diagnosed with CSCR in one of their eyes. All three patients were observed in the initial encounter. One patient was lost to follow-up after the initial visit. The second was observed for 2 months and had improved vision upon the final follow-up (initial VA 20/125 OD, 20/200 OS; final VA 20/80 OD, 20/160 OS). The third was observed for 33 months and maintained his vision from his initial visit (initial VA 20/30 OD, 20/20 OS; final VA 20/25 OD, 20/20 OS). Nine eyes (in six patients) also had intermediate uveitis when diagnosed with CSCR. Two eyes in six patients were treated with focal laser and the rest of the eyes were observed. All nine eyes either maintained or improved their visual acuity in the final follow-up visit.

## 5. Discussion

Our study showed that the most common coexisting ocular comorbidity in the subject eye was non-proliferative diabetic retinopathy (NPDR), with a prevalence of 26.8%, which corresponds with the high rates previously observed in the large-scale CSCR study [4]. There is a noted overlap between features seen in both NPDR and CSCR, including retinal thickening with an intraretinal cyst and subretinal fluid. There also appears to be a change in the pathology of an eye that has both diseases. A cross-sectional study which examined eyes with coexisting NPDR and CSCR found a statistically significant loss of inner segment–outer segment integrity, photoreceptor footplates loss and dilated choroidal vessels in eyes with both diseases, when compared to the fellow eyes with just NPDR [8]. The second most common macular coexisting ocular comorbidity in the study was non-exudative AMD (7.6%). Similarly to a previous study, subject eyes were more likely (OR 2.06, *p*-value = 0.1) to have non-exudative AMD when compared to fellow eyes without CSCR [4].

Regarding the odds, the analysis shows that BRVO was present as a comorbidity in 2.9% of the patients and has a clinically significant OR of 11.56 in subject eyes (*p*-value = 0.02). Previously, it was known that patients with CSCR have an increased chance of retinal vein occlusion (RVO). In 2016, Chang et al. showed that there was a significantly higher risk of RVO in CSCR patients when compared to control patients (3.07 RR, 95% CI, 1.86 to 5.07) [10]. However, Chang et al. (2016) did not specify whether RVO occurred in the same eye as the CSCR (subject eye) or occurred in the fellow eye without CSCR. For all of the comorbidities in the study, our analysis shows the OR for the subject and fellow separately. For BRVO, there is an increased OR of 11.56 (95% CI, 1.55 to 86.1, *p*-value = 0.017) of occurring in the subject eye when compared to fellow eyes. Potential systemic risk factors contribute to the chances of CSCR and BRVO, including the common risk factor of hypertension. However, since systemic factors should affect both eyes, there may be additional factors at play, including proposed local inflammatory processes that may explain the relationship between the two diseases [10].

Similarly, ORs that were not significant in our study can also reveal information regarding the comorbidities in patients with CSCR. For example, a previous study showed that patients with CSCR have an increased 7.85 adjusted hazard ratio (95% confidence interval 4.75–12.97) of having rhegmatogenous retinal detachment (RRD) [11]. That study did not specify on whether the subject or fellow eye had increased RRD. Our study shows a clinically non-significant OR (*p* = 0.58) of RRD occurring between the subject and fellow eyes. A possible explanation for the non-statistically significant risk of RRD in either eye could be the presence of lattice degeneration, a known risk factor for RRD. Lattice degeneration was the most common non-macular ocular comorbidity present in the subject eyes (8.9%, OR 1.06, *p*-value = 0.79), and it presented with similar frequency (8.4%) in the fellow eyes. Patients with CSCR present with increased lattice degeneration in both eyes; therefore, they are at risk for RRD in both the subject and fellow eyes [12].

Examining of simultaneous ocular comorbidities is helpful in the identification and management of CSCR. While there are often times that the diagnosis and treatment of CSCR is straightforward, the presentation can be complicated when it is not present with its classic characteristics or it is non-responsive to conventional treatments [13]. Conditions that mimic CSCR include idiopathic polypoidal choroidal neovascularization (IPCV), macular telangiectasia (MacTel) and posterior uveitis. As part of the differential for any of the beforementioned diseases, the possibility of coexistent CSCR in the same eye must also be considered.

MacTel is a common imitator of CSCR. However, CSCR and MacTel have been reported to present in the same eye in case reports [6,14]. It has a prevalence of 0.1% in the general population [7]. In our study, 1.6% of eyes had concurrent CSCR and MacTel; there was not a statistically significant OR for the subject eye when compared to the fellow eye. Similarly, the pachychoroid spectrum of disease is defined by increased choroidal thickness, dilation of the outer choroidal vessels and attenuation of Sattler’s layer. Ref. [15] Included in this spectrum are CSCR and ICPV. Both diseases also share type 1 choroidal neovascularization, which makes diagnosis of these diseases difficult. However, the coexistence of CSCR and PCV has been previously reported by Manayath [9]. In our study, there appears to be a trend towards significant odds (4.43 OR, *p*-value = 0.05) of a subject eye having both diseases when compared to the fellow eyes.

Lastly, CSCR with concurrent uveitis can be a challenge to both diagnose and treat. Diagnostically, posterior inflammatory disorders can mimic CSCR, and thus can be improperly treated with steroids [16]. A retrospective case series of 22,721 patients with uveitis over 10 years in a tertiary eye center in India found a total 31 eyes in 26 patients with both diagnoses [17]. Because uveitis is often treated with oral corticosteroids, 23 patients (88.5%) were on an oral corticosteroid, and two eyes of two patients received a periocular corticosteroid injection when the diagnosis of CSCR was made. From a treatment perspective, steroids are contraindicated in CSCR [18]. None of the patients in our study that had intermediate or posterior uveitis were treated with oral or steroid drops. Through observation alone, all eleven eyes that had more than one visit in the clinic had a good visual outcome.

The strength of this study is the large number of patients included. This provides greater statistical power regarding the concurrence of ocular comorbidities and CSCR. One limitation of our study is that the data were gathered from multiple sites around the world. Based on the patient population, prevalence of certain ocular comorbidities may vary in certain regions. Additionally, there are limitations due to the retrospective nature of the study, which relies on appropriate classification and documentation of the findings. The ocular comorbidities may predate, be concurrent with or follow the diagnosis of CSCR. Future direction for this study would be to stratify CSCR into acute versus chronic subtypes and find its association with ophthalmology comorbidities.

In conclusion, our large database study shows 6.32% of coexistent ocular conditions with CSCR, the most common being NPDR. Additionally, certain comorbidities, such as BRVO (OR 11.56, *p* = 0.02) and non-exudative AMD (OR 2.06; *p* = 0.01), have greater odds of presenting in the subject eyes when compared to the fellow eyes. Understanding common ocular associations helps clinicians to develop treatment strategies and better outcomes. Additionally, it can be valuable when evaluating follow-up studies and understanding the treatment outcomes.

## Figures and Tables

**Table 1:** Demographics of the patients.

Age (average ± standard deviation)		50.30 ± 13.09 years	
		Number	Percentage
Sex	Male	465	80.31
	Female	114	19.69
Subject Eyes	Right	204	35.23
	Left	188	32.47
	Both	187	32.3
Baseline VA	Right	0.55 ± 0.31 logMAR	
(average ± standard deviation)	Left	0.59 ± 0.38 logMAR	
	Subject eye	0.49 ± 0.36 logMAR	
	Fellow eye	0.84 ± 0.28 logMAR	
Medical History		Number	Percentage (%)
	Arthritis	6	1.04
	Anxiety	6	1.04
	Asthma	7	1.21
	Benign Prostatic Hyperplasia	1	0.17
	Cancer	2	0.35
	Coronary Artery Disease	11	1.9
	Cardiac Heart Failure	1	0.17
	Crohn’s Disease	1	0.17
	Diffuse Cerebral Atrophy	1	0.17
	Dyslipidemia	20	3.45
	Diabetes Mellitus	202	34.89
	Fibroid Uterus	1	0.17
	HIV	1	0.17
	Hypertension	86	14.85
	Insomnia	2	0.35
	Hypothyroidism	7	1.21
	Multiple Sclerosis	1	0.17
	Obesity	5	0.86
	Obstructive Sleep Apnea	5	0.86
	Osteoporosis	1	0.17
	Non-Alcohol Fatty Liver Disease	1	0.17
	Parkinson Disease	2	0.35
	Peptic Ulcer	1	0.17
	Psoriasis	1	0.17
	Seizure	1	0.17
	Systemic Lupus Erythematosus	8	1.38
	Tuberculosis	1	0.17

Table 1 shows the baseline demographic data of the patients in this study. Subject eyes had at least one other additional comorbidity along with central serous choroidopathy (CSCR). For patients with a presentation of CSCR in only one eye, the other eye was recorded as a fellow eye. For patients with a presentation of CSCR in both eyes, both eyes were recorded as subject eyes.

**Table 2:** Ocular comorbidities in eyes with central serous chorioretinopathy.

		Subject Eyes				Fellow Eyes	
		Number	%	OR (95% CI)	*p*-Value	Number	%
**Glaucoma**							
Angle closure		12	1.6	3.10 (0.69 to 13.94)	0.14	2	0.5
Glaucoma suspected		4	0.5	1.02 (0.19 to 5.61)	0.98	2	0.5
Ocular Hypertension		3	0	1.54 (0.16 to 14.83)	0.71	1	0
Primary Open Angle Glaucoma		6	0.8	0.61 (0.19 to 2.01)	0.42	5	1.3
Pseudoexfoliation Syndrome		1	0	1.5 (0.06 to 37.85)	0.79	0	0
**Optic Nerve**							
Disc Edema—unspecified		7	0.9	1.80 (0.37 to 8.70)	0.77	2	0.5
Non-Arteritic Ischemic Optic Neuropathy		8	1.0	2.06 (0.43 to 9.74)	0.36	2	0.5
Neuroretinitis		4	0.5	4.63 (0.25 to 86.27)	0.30	0	0
Optic atrophy		39	5.1	1.35 (0.73 to 2.48)	0.34	15	3.8
Optic nerve head drusen		2	0	2.6 (0.12 to 53.6)	0.54	0	0
Optic neuritis		12	1.6	4.63 (0.60 to 35.74)	0.14	1	0.3
Optic neuropathy—non-specific		1	0	1.5 (0.06 to 37.85)	0.79	0	0
Optic pit		13	1.7	3.37 (0.76 to 14.99)	0.11	2	0.5
Papillitis		2	0.3	2.57 (0.12 to 53.60)	0.54	0	0
**Findings**							
Asteroid Hyalosis		9	1.2	9.84 (0.57 to 169.60)	0.11	0	0
Congenital hypertrophy of the retinal pigment epithelium		4	0.5	1.02 (0.19 to 5.61)	0.98	2	0.5
Choroidal folds *		3	0.4	1.54 (0.16 to 14.83)	0.71	1	0.3
Disseminated choroiditis		5	0.7	1.28 (0.25 to 6.63)	0.77	2	0.5
Epiretinal Membrane *		25	3.3	1.07 (0.53 to 2.15)	0.85	12	3.1
Focal retinitis		1	0	1.5 (0.06 to 37.85)	0.79	0	0
Lattice degeneration		68	8.9	1.06 (0.69 to 1.64)	0.79	33	8.4
Macular hole *		7	0.9	3.61 (0.44 to 29.41)	0.23	1	0.3
Pseudo macular hole *		1	0.1	1.54 (0.06 to 37.85)	0.79	0	0
Nevus		14	1.8	15.13 (0.90 to 254.25)	0.06	0	0
Neuroretinitis		4	0.5	4.63 (0.25 to 86.27)	0.30	0	0
Pavingstone Degeneration		5	1	1.28 (0.25 to 6.63)	0.77	2	1
Peripheral Pigmentary changes		26	3	2.72 (1.04 to 7.14)	**0.03**	5	1
Peripheral retinal breaks							
	Dialysis	1	0.1	1.54 (0.06 to 37.85)	0.79	0	0
	Holes	4	1	0.68 (0.15 to 3.06)	0.62	3	0.8
	Tears	4	0.5	0.51 (0.13 to 2.05)	0.34	4	0.5
Retinal perivasculitis		1	0	1.5 (0.06 to 37.85)	0.79	0	0
Retinoschisis		4	0.5	4.63 (0.25 to 86.27)	0.30	0	0
Rhegmatogenous Retinal Detachment		13	1.7	1.34 (0.47 to 3.78)	0.58	5	1.3
Vitreous Hemorrhage		10	1.3	1.72 (0.47 to 6.27)	0.41	3	0.8
**Retinal Disease**							
Age-Related Macular Degeneration *	Exudative	9	1.2	9.84 (0.57 to 169.60)	0.12	0	0.11
	Non-Exudative	58	7.6	2.06 (1.15 to 3.68)	**0.01**	15	3.8
Best’s disease		4	0.5	4.63 (0.25 to 86.27)	0.30	0	0
Branch Retinal Artery Occlusion *		2	0.3	2.57 (0.12 to 53.60)	0.54	0	0
Branch Retinal Vein Occlusion *		22	2.9	11.56 (1.55 to 86.10)	**0.02**	1	0.3
Central Areolar Dystrophy *		2	0.3	2.57 (0.12 to 53.60)	0.54	0	0
Central Retinal Artery Occlusion *		4	0.5	4.63 (0.25 to 86.30)	0.30	0	0
Central Retinal Vein Occlusion *		5	0.7	5.67 (0.31 to 102.80)	0.24	0	0
Coat’s disease		1	0.1	1.54 (0.06 to 37.85)	0.79	0	0
Cone dystrophy *		4	0.5	2.05 (0.23 to 18.43)	0.52	1	0.3
Diabetic Retinopathy *	Non-Proliferative	205	26.8	1.14 (0.82 to 1.57)	0.09	87	22.2
	Proliferative	8	1.0	0.82 (0.27 to 2.51)	0.35	5	1.3
Disseminated retinitis and retinochoroiditis *		5	0.7	1.28 (0.25 to 6.63)	0.77	2	0.5
Hypertensive Retinopathy *		23	3.0	1.18 (0.56 to 2.51)	0.66	10	2.6
Ischemic Vasculopathy		2	0	2.6 (0.12 to 53.6)	0.54	0	0
Idiopathic polypoidal choroidal vasculopathy *		17	2.2	4.43 (1.02 to 19.25)	**0.05**	2	0.5
Macular telangiectasia *		12	1.6	1.02 (0.38 to 2.75)	0.96	6	1.5
Melanoma		1	0.1	1.54 (0.06 to 37.85)	0.79	0	0
Multifocal choroiditis *		5	0.7	2.57 (0.3 to 22.07)	0.39	1	0.3
Myopic choroid neovascular membrane *		1	0.1	1.54 (0.06 to 37.85)	0.79	0	0
Punctate Inner Choroiditis *		3	0.4	1.54 (0.16 to 14.83)	0.71	1	0.3
Retinitis Pigmentosa *		6	0.8	1.54 (0.31 to 7.66)	0.60	2	0.5
Rod Dystrophy *		1	0	0.51 (0.03 to 8.19)	0.63	1	0
Serpiginous choroiditis *		2	0.3	0.51 (0.07 to 3.64)	0.50	2	0.5
Stargardt’s disease *		6	0.8	6.71 (0.38 to 119.41)	0.20	0	0
Sympathetic Ophthalmoplegia *		1	0.1	0.51 (0.03 to 8.19)	0.64	1	0.3
Toxic Retinopathy *		2	0.3	0.51 (0.07 to 3.64)	0.50	2	0.5
Toxoplasma		1	0.1	1.5 (0.06 to 37.85)	0.79	0	0
Tuberculosis granuloma		4	0.5	4.63 (0.24 to 86.27)	0.30	0	0
Vasculitis—non-specific		4	1	1.02 (0.19 to 5.61)	0.98	2	1
Vitelliform macular dystrophy *		12	1.6	6.22 (0.81 to 48.03)	0.08	1	0.3
		**Subject Eyes**				**Fellow Eyes**	
		**Number**	**%**	**OR (95% CI)**	***p*-Value**	**Number**	**%**
**Other Disease**							
Cavernous Fistula		0	0	0.17 (0.01 to 4.19)	0.28	1	0
Coloboma *		4	0.5	0.51 (0.13 to 2.05)	0.34	4	1.0
Posterior Scleritis *		1	0	0.51 (0.03 to 8.19)	0.63	1	0
Uveitis	Anterior	1	0	1.5 (0.06 to 37.85)	0.79	0	0
	Intermediate	10	1.3	2.58 (0.56 to 11.83)	0.80	2	0.2
	Non-specific	3	1	0.77 (0.13 to 4.61)	0.77	2	1
	Panuveitis	3	0.4	0.51 (0.1 to 2.54)	1.59	3	0.8

Table 2: Ocular comorbidity in eyes. In all, 766 of the subject eyes had at least one other additional comorbidity along with central serous choroidopathy (CSCR). There were 392 fellow eyes without CSCR in the study. For patients with a presentation of CSCR in only one eye, the other eye without CSCR was recorded as a fellow eye. For patients with a presentation of CSCR in both eyes, both eyes were recorded as subject eyes. The asterisk (*) denotes ocular comorbidities that were denoted as macular diseases in this study. OR and CI were calculated for CSCR and each disease in the subject eyes. A non-paired two-tailed student’s *t*-test was used to calculate the *p*-value of each comorbidity. A value of < 0.05 was considered statistically significant. OR: odds ratio; CI: confidence interval; %: percentage.

## Data Availability

Not available.
